# Comparative Analysis of Crosslinking Methods and Their Impact on the Physicochemical Properties of SA/PVA Hydrogels

**DOI:** 10.3390/ma17081816

**Published:** 2024-04-15

**Authors:** Konrad Niewiadomski, Daniel Szopa, Katarzyna Pstrowska, Paulina Wróbel, Anna Witek-Krowiak

**Affiliations:** Faculty of Chemistry, Wroclaw University of Science and Technology, 27 Wybrzeze Wyspianskiego Street, 50-370 Wroclaw, Polanddaniel.szopa@pwr.edu.pl (D.S.); 252570@student.pwr.edu.pl (P.W.)

**Keywords:** alginate, polyvinyl alcohol, crosslinking, hydrogels, composites

## Abstract

Hydrogels, versatile materials used in various applications such as medicine, possess properties crucial for their specific applications, significantly influenced by their preparation methods. This study synthesized 18 different types of hydrogels using sodium alginate (SA) and two molecular weights of polyvinyl alcohol (PVA). Crosslinking agents such as aqueous solutions of calcium (Ca^2+^) and copper (Cu^2+^) ions and solutions of these ions in boric acid were utilized. The hydrogels were subjected to compression strength tests and drying kinetics analysis. Additionally, six hydrogel variants containing larger PVA particles underwent Fourier-transform infrared spectroscopy (FTIR) and thermogravimetric analysis (TGA) post-drying. Some samples were lyophilized, and their surface morphology was examined using scanning electron microscopy (SEM). The results indicate that the choice of crosslinking method significantly impacts the physicochemical properties of the hydrogels. Crosslinking in solutions with higher concentrations of crosslinking ions enhanced mechanical properties and thermal stability. Conversely, using copper ions instead of calcium resulted in slower drying kinetics and reduced thermal stability. Notably, employing boric acid as a crosslinking agent for hydrogels containing heavier PVA molecules led to considerable improvements in mechanical properties and thermal stability.

## 1. Introduction

The manufacture of plastics has significantly contributed to global environmental pollution. With the continual accumulation of plastic waste, an urgent demand for sustainable alternatives arises. Biodegradable polymers present a promising solution to this issue by reducing pollution and lessening environmental impact. Utilizing innovative technologies and manufacturing processes allows for the development of materials that adhere to stringent quality and performance standards while promoting ecological sustainability. Hydrogels, versatile polymeric materials, find diverse applications across various fields. In medicine, their primary role lies in wound care [1], particularly for challenging cases like diabetic foot ulcers [2]. They serve as effective scaffolds for tissue growth [2]. In cosmetics, they are utilized in products that preserve skin moisture [3]. The food industry employs hydrogels as nutrient carriers, components of packaging, and food freshness sensors [4,5,6]. In agriculture, they function well in irrigation formulations and targeted nutrient delivery [7,8]. A crucial characteristic is their capacity to regulate ingredient release, facilitated by their porous structure. The interconnections of polymer chains within hydrogels can be adjusted based on network packing density and interaction between components. Biodegradable hydrogels emerge as a particularly appealing option due to their natural origins and ability to degrade without releasing harmful substances into the environment. These hydrogels, characterized by their capacity to retain water, consist of two- or multi-component systems wherein synthetic and natural polymers serve as essential building blocks. Intertwining polymer chains form intricate three-dimensional networks capable of entrapping dispersed water, depending on the crosslinking agents used.

Sodium alginate (SA) is a natural polysaccharide obtained as a byproduct during the extraction of mannitol and iodine from kelp or Sargassum in brown algae. Its molecular structure comprises β-mannuronic acid (M) and α-guluronic acid (G) linked by (1 → 4) bonds. This long-chain polymer consists of GM, MM, and GG segments in specific proportions. Depending on the content of the G and M segments, formed gels can be more or less brittle because G segment reacts with divalent cations to form gels [9]. Sodium alginate is extensively utilized due to its linear structure, sensitivity to pH, solubility in water, non-toxicity, biodegradability, hydrophilicity, biocompatibility, thermal stability, safety, and lack of immunogenicity. Furthermore, it possesses chelating abilities, cost-effectiveness, transparency, ease of gelation, mucoadhesive properties, thickening capacity, and film-forming characteristics [10]. These diverse attributes make this polysaccharide highly promising in numerous fields, including food science, drug and gene delivery systems, tissue engineering, wound management, and wastewater treatment [11].

Poly(vinyl alcohol) (PVA) is a synthetic, widely used, non-toxic, water-soluble, chemically stable, biodegradable, and biocompatible hydrogel polymer [12]. PVA hydrogel can be prepared using both chemical and physical methods. The chemical method involves using multifunctional aldehyde molecules (such as glutaraldehyde, glyoxal), species containing boron, and irradiation techniques. However, physically crosslinked PVA hydrogels are preferred because of their high purity and easy preparation. This method includes repetitive freezing–thawing cycles leading to the structural change of PVA into a gel and is based on intermolecular interactions such as hydrophobic interactions, ionic or electrostatic interactions, and hydrogen bonding [13,14]. PVA-based hydrogels have considerable potential to become a component in human body sensors, triboelectric nanogenerator sensors, and human–computer interaction devices [12]. Moreover, they can be used in other biomedical applications like cell culture, tissue engineering, and drug delivery [15].

However, hydrogels are characterized by low mechanical strength, rendering them impractical for many applications. Additives such as nanocellulose, proteins, and polysaccharides can be incorporated without compromising valuable features like biodegradability or cost-effectiveness to improve their mechanical properties. Furthermore, these additives can expand the range of potential hydrogel applications [16]. Another method to strengthen the mechanical properties of SA/PVA hydrogels is using a combination of processes of salting out and freezing–thawing cycles [17]. Double network structures of hydrogels also improve stretchability toughness. They are constructed of two types of crosslinked single networks with different properties. The enhanced mechanical properties of dual interpenetrating networks most likely stem from stress transfer between the doubly crosslinked chains [18].

This article details the preparation of 18 varieties of hydrogel composites utilizing an SA/PVA interpenetrating network. The hydrogels underwent crosslinking through various methods, including the use of divalent ions such as Ca^2+^ and Cu^2+^ at varying concentrations, borate ions, and mixed solutions containing metal ions in saturated boric acid. Calcium ions are utilized in alginate crosslinking for their biocompatibility and ability to form stable gel networks, while copper ions are employed for their antimicrobial properties and promotion of wound healing, making them valuable in applications requiring infection control and tissue regeneration. These diverse crosslinking methods lead to the formation of distinct connections within the gel structure, thereby influencing the properties of the polymer network. A comprehensive analysis of the resulting hydrogels was conducted, including mechanical strength, thermal stability, and morphological characterization of the beads.

## 2. Materials and Methods

### 2.1. Reagents

Reagents in the form of polymer solutions (sodium alginate, PVA (named PVA1) 13,000–23,000 g/mol and 30,000–70,000 g/mol (named PVA2) were purchased from Sigma-Aldrich, St. Louis, MO, USA) were used to prepare the hydrogel composites. For hydrogel crosslinking, CaCl_2_, CuSO_4_, and boric acid salts purchased from POCH (Gliwice, Poland) were used.

The base polymer mixtures were prepared at their highest achievable concentrations (SA 4%, PVA1 2%, and PVA2 10%). Calcium chloride and copper(II) sulfate solutions were prepared at 0.2 M and 0.5 M concentrations, respectively. Boric acid was dissolved in heated distilled water to form a saturated solution. Calcium chloride and copper(II) sulfate were also prepared using a solution of H_3_BO_3_ as the solvent. Finally, sodium alginate, PVA1, and PVA2 were combined appropriately to form base mixtures to prepare hydrogels (Table 1).

### 2.2. Preparation of Hydrogels

Eighteen composites based on different blends of polymers and crosslinking agents were prepared (Table 2). The polymer mixtures were dropped into a suitable crosslinking agent to form spherical structures. The structures formed immediately, but were left to crosslink completely. After an hour, once the hydrogel beads had formed, they were drained using a sieve and gently dried with paper towels. Hydrated hydrogels were stored in the fridge in a tightly sealed container.

### 2.3. Examination of Mechanical Properties

A constant head speed of 6 mm/min was maintained using an INSTRON 5966 machine (INSTRON,Norwood, MA, USA) to assess the compressive strength of the hydrogels. The maximum compressive strength was identified upon hydrogel cracking, with a threshold of applied force change exceeding 5%. The Young’s modulus was calculated for each hydrogel variant, along with values for maximum compressive force (F_max_), and the corresponding deformation and displacement of the test sample at F_max_ were documented. Before each measurement was taken, the diameter of the sample was measured with a caliper and entered into the program. Each type of hydrogel underwent ten repetitions for strength measurements. The diameter of all hydrogel samples was approximately 5 mm.

### 2.4. Drying Kinetics

To ascertain the moisture content, samples with known mass were weighed at 30 min intervals for 3 h, with excess water removed using paper each time. This process was repeated after 6, 12, 24, and 30 h, allowing the samples to air dry at room temperature. The study was conducted in duplicate, and the averages of the measurements were used for graphical analysis. The drying kinetics were analyzed using two mathematical models, the Newton (Equation (1)) and the Page model (Equation (2) [7]). The moisture content ratio was also determined (Equation (3) [7]).

(1) MR=exp ⁡(−kt)

(2) $$MR=\exp\;(-kt^{n})$$

(3) $$MR=\frac{X-X_{e}}{X_{0}-X_{e}}$$

Six dried hydrogels ((AP2_Ca_02, AP2_Ca_05, AP2_Ca_B, AP2_Cu_02, AP2_Cu_05, and AP2_Cu_B) were chosen based on visual inspection for further analysis using FTIR and TGA. These same hydrogels underwent lyophilization and were observed via SEM.

### 2.5. FTIR Analysis

The dried hydrogel samples containing PVA2 underwent Fourier-transform infrared spectroscopy (FTIR) analysis, performed using an IRAffinity-1S spectrometer (Shimadzu, Kyoto, Japan) equipped with an ATR Specac Quest (transmittance mode, 32 scans, and 400–4000 cm^−1^ wavelength range).

### 2.6. Thermogravimetric Analysis

The same set of samples used for FTIR analysis underwent TGA analysis. Each hydrogel sample was loaded into a ceramic crucible within the chamber of the 5E-MAC6710 thermogravimetric analyzer (Changsha Kaiyuan Instruments Co., Changsha, China), and subjected to heating from room temperature to 850 °C at a constant rate of 2 °C/min, under an inert atmosphere of pure (99.9%) nitrogen with a flow rate at a level of 5 L/min.

### 2.7. SEM Observation

Observations via scanning electron microscopy were performed on hydrogels containing PVA2 that had undergone lyophilization. A thin layer of gold was applied using a vacuum sputter coater to prepare the lyophilized samples for observation. The SEM/GA-FIB FEI HELIOS NANOLAB 600I microscope (Hillsboro, OR, USA), was employed for this purpose. Images were captured at magnifications ranging from 74 to 10,000, typically 8–24 images/sample, focusing on identifying the characteristic features. The chemical composition of the samples was determined semi-quantitatively using energy dispersive spectroscopy (EDS) with a Quanta 250 scanning electron microscope at 15 kV.

## 3. Results

### 3.1. Visual Analysis of Hydrogels

**Figure 1:**
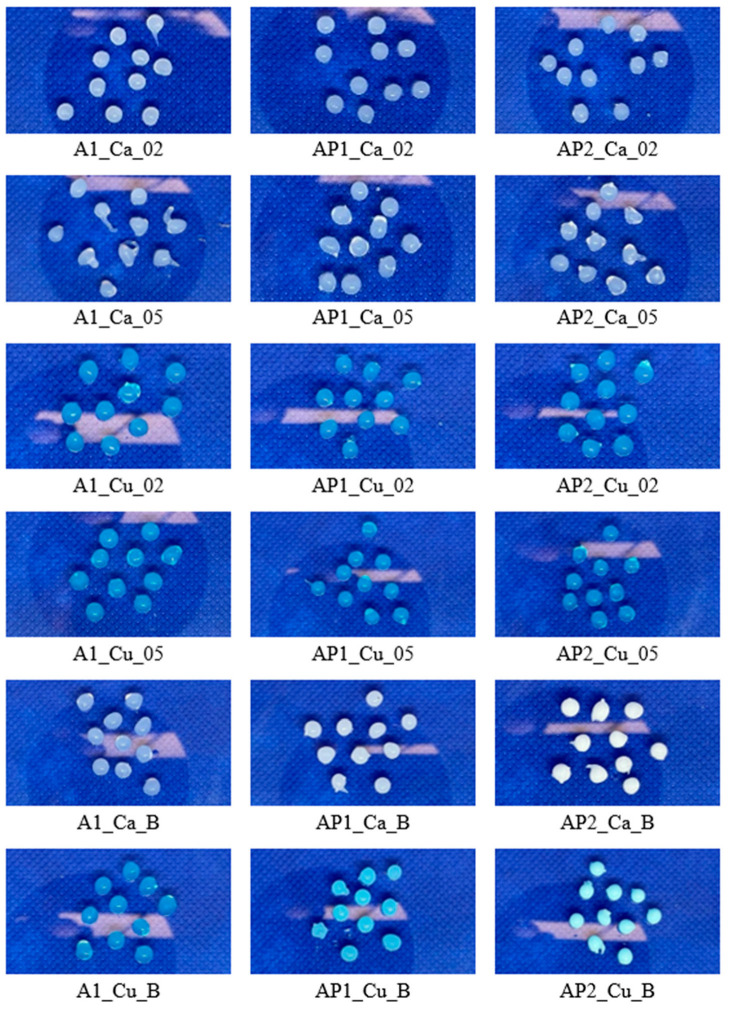
Photographs of the analyzed hydrogel types.

The acquired structures were subjected to qualitative assessment regarding their structure, shape, and color, as presented in Figure 1. Most of the matrices were spherical, where higher crosslinking agent concentrations caused structural deformations. This is notable for crosslinking with calcium ions (A1_Ca_05, AP2_Ca_05). Crosslinking with higher concentrations of Cu^2+^ ions did not result in significant deformations of the hydrogels. Applying 0.2 M and 0.5 M Cu^2+^ resulted in the blue color of the acquired spherical structures with slight tear-like distortion. Hydrogels crosslinked with Ca^2+^ ions had a milky, transparent color. The addition of H_3_BO_3_ did not cause significant changes for matrix A1. However, by adding PVA, a color change for both crosslinking methods (Ca^2+^, Cu^2+^) was observed as the samples became milky and unclear. The shape itself did not undergo significant visual changes. Notably, sample AP1 containing 2% PVA1 showed a greater tendency to maintain a spherical shape. This property is evident when comparing the 0.5 M Ca^2+^ crosslinked group.

Alginate-based hydrogels primarily comprise polymer chains consisting of alginate residues [19]. Adding divalent ions like Ca^2+^ or Cu^2+^ to the aqueous solution leads to bonding cations to the carboxyl anions, forming branched networks [20]. These networks maintain the material’s structure and facilitate the retention of the solution within. Polyvinyl alcohol (PVA), as a polymer comprising non-dissociating molecules, does not possess the capability to link its chains in the same manner as alginate does through calcium and copper ions. Nonetheless, it can be incorporated into Alg-Ca or Alg-Cu network structures, thus impacting the physical and chemical properties of the final product. When hydrogel formation occurs via crosslinking in the SA-PVA solution using divalent ions, it forms a semi-interpenetrating polymer network, where the PVA chains remain unbound to the rest of the structure [21]. Furthermore, the properties of the hydrogel obtained vary depending on the size distribution of the PVA molecules used, as indicated by the findings presented in the preceding chapter. One plausible approach to chemically crosslinking PVA molecules involves using boric acid. In a PVA solution, borate ions attach to pairs of adjacent hydroxyl groups within one polymer molecule and then to two adjacent hydroxyl groups in another chain, initiating a reaction known as diol complexation [22]. Hydrogels crosslinked using this method are expected to exhibit a high degree of particle packing and numerous bonds.

### 3.2. Examination of Mechanical Properties

**Figure 2:**
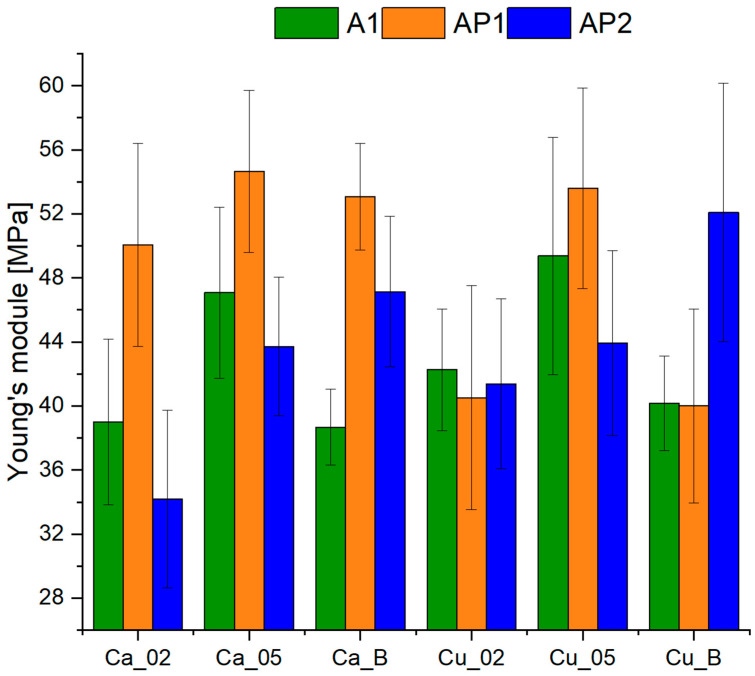
Young’s modulus values of hydrogels for individual crosslinking media and polymer blends.

The comparison of mechanical test results is presented in Figure 2. The lowest Young’s modulus values were acquired by utilizing the A1 polymer mixture, where an increase occurred by crosslinking with 0.5 M compounds compared to 0.2 M. Improvement in mechanical parameters was observed for the AP2 matrix with the addition of 10% PVA2. The modulus value increased due to higher Ca^2+^ and Cu^2+^ ion concentrations for this combination. In both cases, adding H_3_BO_3_ resulted in a significant increase in Young’s modulus value compared to crosslinking methods without boron ions. A similar trend is present when using lower concentrations of PVA; in this case, AP1 with 2% PVA1 allowed for the most satisfactory mechanical parameters to be achieved with a Young’s modulus of approximately 54 MPa. Adding H_3_BO_3_ in crosslinking with Cu^2+^ ions significantly deteriorated this parameter compared to a 0.5 M solution. Similar results were achieved using Ca^2+^ in the three investigated cases, but the highest value was achieved when applying 0.2 M containing Ca^2+^ ions.

Structures without the PVA addition revealed a higher Young’s modulus value with increased Ca^2+^ ion concentration, consistent with the available literature [23]. The same trend can be observed during the usage of Cu^2+^ ions. This suggests that increased concentration led to greater bonding with alginate, facilitating the integration of PVA particles into the hydrogel network and enhancing its mechanical characteristics. This enhancement arises from developing a more intricate and condensed hydrogel structure, facilitating a more efficient distribution of applied force. The PVA1 addition significantly increased the Young’s modulus value for hydrogels where Ca^2+^ ions were used, indicating the formation of additional bonds in the hydrogel structure. As for the usage of Cu^2+^ ions, the increase was only observed with the use of a higher concentration of 0.5 M. It is likely that the formed bonds are capable of trapping PVA particles in the network, where the structure crosslinked with a 0.2 M solution undergoes cracking [24]. With the application of PVA2, an increase in the concentration of Cu^2+^ ions led to a significant decrease in the strength of the acquired hydrogels. This may be related to forming more compact networks than those with Ca^2+^ ions, resulting in larger PVA particles negatively impacting crosslinking. Crosslinking with Ca^2+^ ions showed an expected trend of increasing Young’s modulus with increasing concentration. However, it is worth noting that adding H_3_BO_3_ improves the structure’s strength, indicating the formation of comprehensive diol bonds. It is probable that the presence of complex bonds between boron ions and PVA significantly impacts the mechanical properties and aids in network formation, particularly with larger particles. It is important to highlight that utilizing boric acid for SA and PVA1 did not enhance strength. The addition of boron ions causes the reaction of hydroxyl groups contained in the sodium alginate and PVA, which translates into the formation of cross-links between polymers [25,26]. This leads to a decelerated gelation process, yielding a tighter and more stable structure. In the case of resistance tests, we see that the addition of PVA improved the mechanical properties, but the use of higher PVA concentrations (PVA2) resulted in a decrease compared to PVA1. This is due to an excessively high PVA concentration. Despite being an elastic polymer, an excess of it as an additive increases the stiffness and brittleness of the hydrogel. Because of its association with sodium alginate, an excess of PVA might hinder the complete crosslinking of sodium alginate by competing for water, thereby diminishing the adhesion between polymer particles [27].

### 3.3. SEM Observation

**Figure 3:**
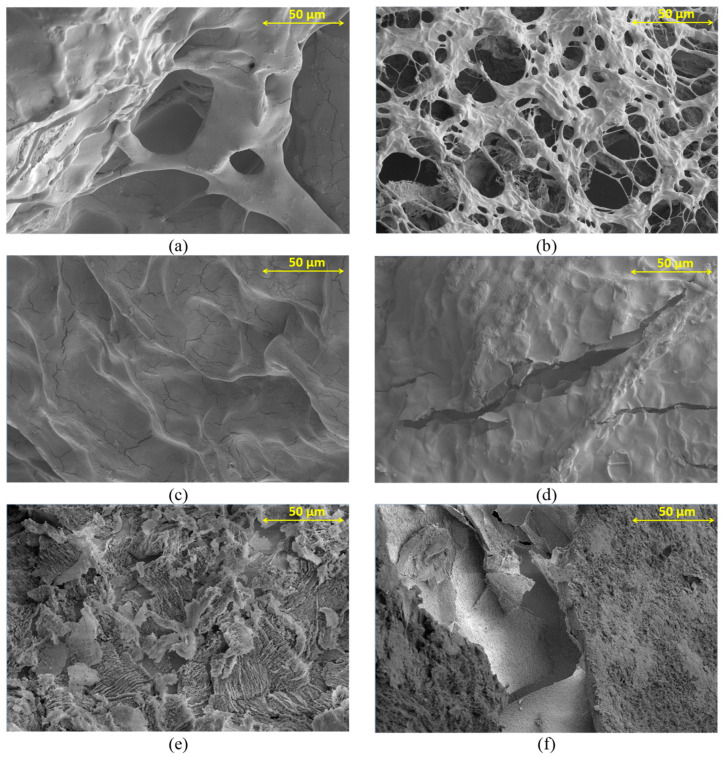
SEM analysis magnification 1000×: (**a**) AP2_Ca_02; (**b**) AP2_Cu_02; (**c**) AP2_Ca_05; (**d**) AP2_Cu_05; (**e**) AP2_Ca_B; (**f**) AP2_Cu_B.

**Figure 4:**
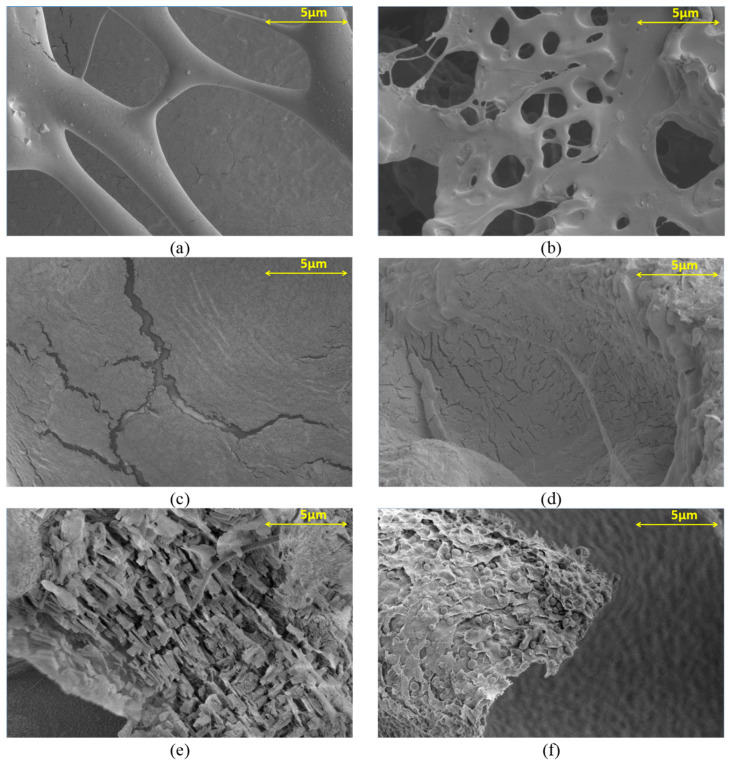
SEM analysis magnification 10,000×: (**a**) AP2_Ca_02; (**b**) AP2_Cu_02; (**c**) AP2_Ca_05; (**d**) AP2_Cu_05; (**e**) AP2_Ca_B; (**f**) AP2_Cu_B.

The magnified approximations of the structures for AP2 are presented at 1000× (Figure 3) and 10,000× (Figure 4). In both photographs, (Figure 3a,b) depict the hydrogel structure when using Ca^2+^ and Cu^2+^ ions at a concentration of 0.2 M, respectively. Loose crosslinking with numerous free spaces is visible. The structures possess a similar framework, which, for the Cu^2+^ ions, is more packed with fewer pores. Photographs in Figure 3c,d demonstrate the degree of crosslinking when using a concentration of 0.5 M. Significant improvement in structure crosslinking is evident, resulting in a denser, more uniform surface, although cracks in the structure are still visible. Cracks are more apparent in the hydrogel crosslinked with Ca^2+^ ions. Crosslinking with the boric acid addition is displayed in Figure 3e,f. The surfaces are much denser than those without boron ions in Figure 3a,b, but their structure has a rough surface with numerous indentations, indicating a high degree of layering in the resulting hydrogel structures.

By analyzing the structure of acquired hydrogels in Figure 3 and Figure 4, significant differences can be observed from the applied crosslinking method. When comparing the influence of crosslinking ion concentration, it can be noted that increasing the concentration of Ca^2+^ ions from 0.2 M (Figure 4a) to 0.5 M (Figure 4c) resulted in an increase in the bond quantity with sodium alginate and thus decrease in the number of the pores [28]. This indicates a better crosslinking quality, which is also evident for samples crosslinked with Cu^2+^ ions (Figure 4b,d). When examining the utilization of specific ions, it becomes evident that using Cu^2+^ ions results in more densely packed structures with smaller pores than those formed when Ca^2+^ ions are used. It is particularly apparent when comparing the structure for 0.5 M crosslinking solutions (Figure 4c,d). This indicates improved stability of the obtained structures and better crosslinking efficiency, resulting in the strength of the obtained structures [29]. The difference in both crosslinking methods results from differences in the bonds formed by Ca^2+^ ions, which have a greater tendency to form bonds with the carboxyl groups contained in sodium alginates. Yet, Cu^2+^ ions tend to bind with the hydroxyl groups contained in PVA. The properties of hydrogel structures made from sodium alginate and PVA confirm the effects of Cu^2+^ ions. These hydrogels act as ideal adsorbents, and their adsorption properties increase with smaller crosslinking of the structure. After adsorption of Cu^2+^ ions by the hydrogel, the structure increased its mechanical properties and the crosslinking degree [30]. For this reason, in matrices containing a higher PVA concentration (AP2), the structure containing Cu^2+^ ions has a more compact structure than crosslinking with Ca^2+^ ions. Moreover, Ca^2+^ ions tend to form more flexible structures, while hydrogels crosslinked with Cu^2+^ ions are more rigid. This distinction is depicted in Figure 2, where hydrogels crosslinked with Cu^2+^ ions display increased Young’s modulus values, particularly in AP2 structures with the highest PVA content. A reverse trend is visible in structures without PVA and with a lower PVA concentration [31]. However, hydrogels crosslinked with the addition of H_3_BO_3_ exhibited significant structural continuity without visible large pores. As intended, the H_3_BO_3_ presence resulted in the formation of complex diol bonds with PVA, which improved the structure of the hydrogel. These bonds directly increase mechanical strength by reducing free spaces in the material structure [32].

**Figure 5:**
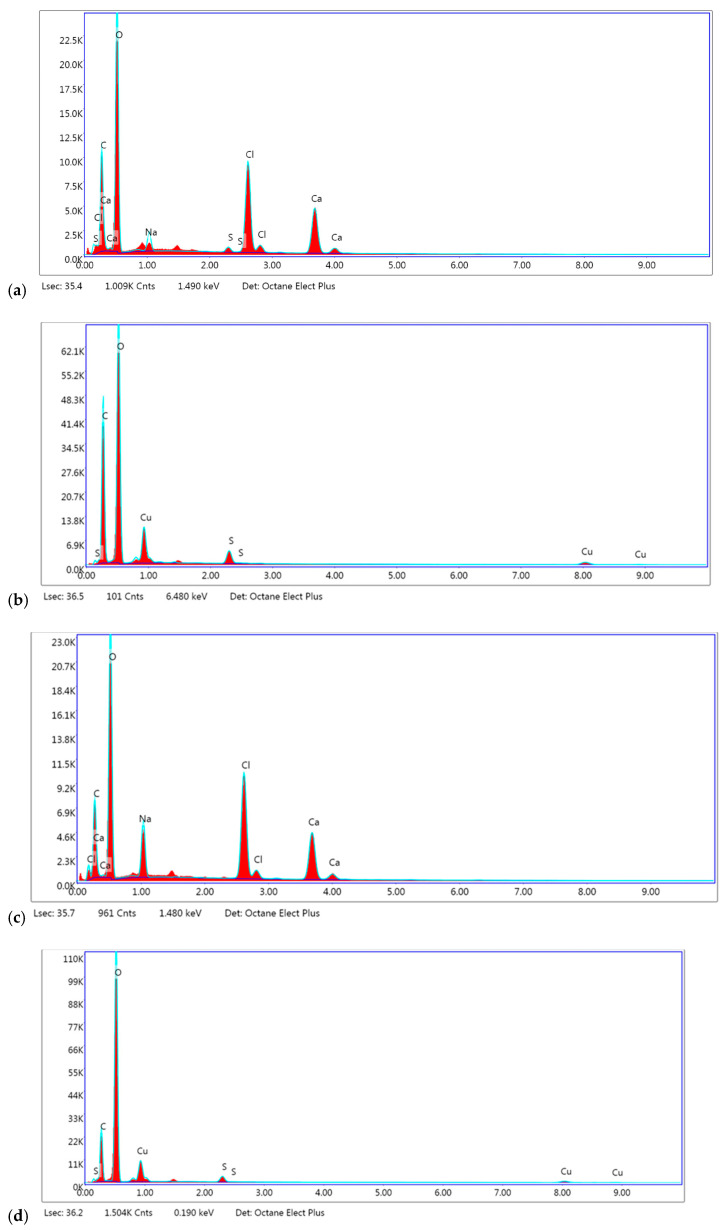
SEM-EDS analysis of samples: (**a**) AP2_Ca_02, (**b**) AP2_Cu_02, (**c**) AP2_Ca_B, (**d**) AP2_Cu_B.

Based on the SEM-EDS analysis, the calcium content of the samples crosslinked with calcium ions is in the range of 10.65–11.42 wt%, while for the samples crosslinked with copper ions, the percentage of this element is 6.44–7.11% (Figure 5). The proportion of boron ions was impossible to determine by this method due to overlapping peaks with other light elements.

### 3.4. Drying Kinetics

**Figure 6:**
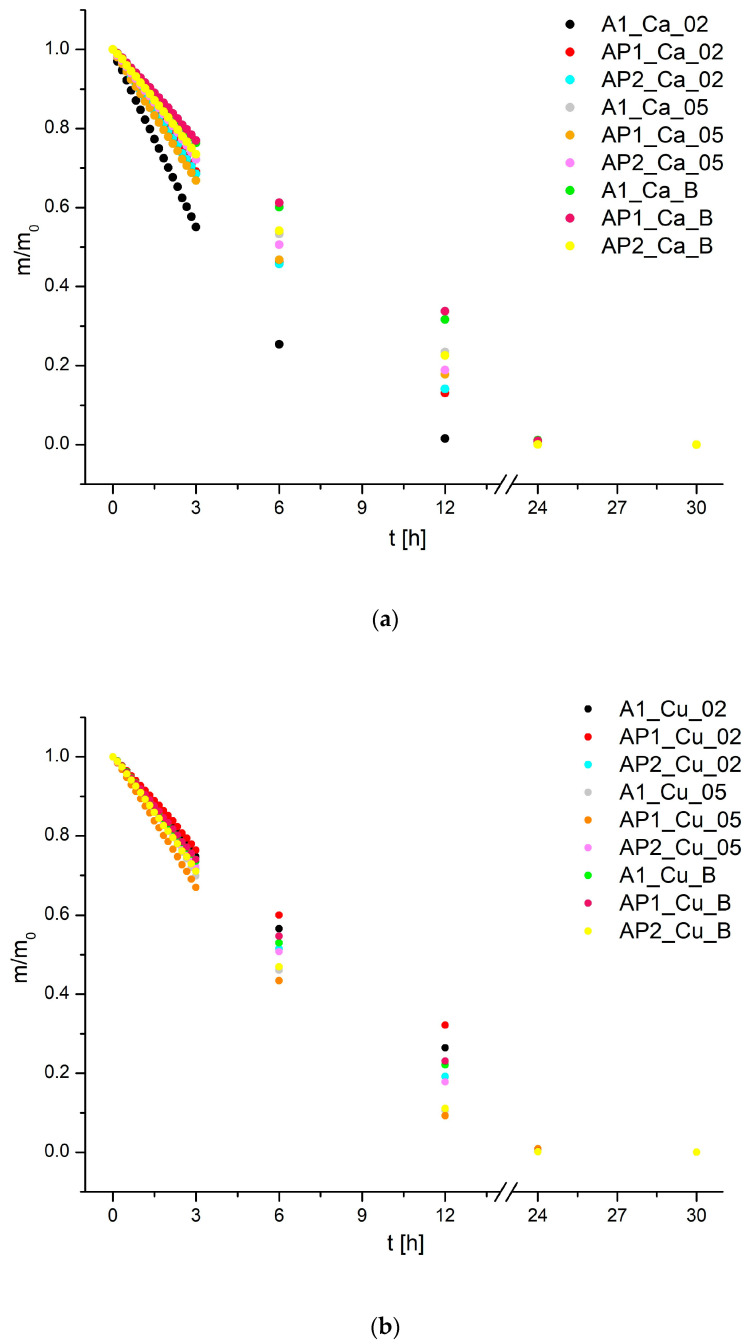
Drying kinetic at room temperature: (**a**) hydrogels crosslinked with Ca^2+^, (**b**) crosslinked with Cu^2+^.

The kinetics study was conducted to check the change in mass of the hydrogels over time, as presented in Figure 6a,b. According to the compiled data, it can be determined that the mass loss trend progressed linearly for each of the hydrogels over time, with the most dynamic mass decrease occurring within 3 h. It was decided that the samples were dried after approximately 30 h. Observing the mass loss, it can be stated that sample A1_Ca_02 underwent the fastest drying compared to the rest of the hydrogels. Yet, the samples containing 2% PVA1 crosslinked with Ca^2+^ ions containing the addition of H_3_BO_3_ dried the slowest. The presence of H_3_BO_3_ in all conducted tests resulted in a decrease in the drying rate of the hydrogels. The addition of 2% PVA1 caused a lowering of the drying rate, but increasing the content to 10% PVA2 resulted in a re-increase. It is worth noting that generally, Cu^2+^ ions in the case of A1 matrices allowed for achieving a lower drying degree, which is particularly noticeable for samples A1_Ca_02 and A1_Cu_02.

**Figure 7:**
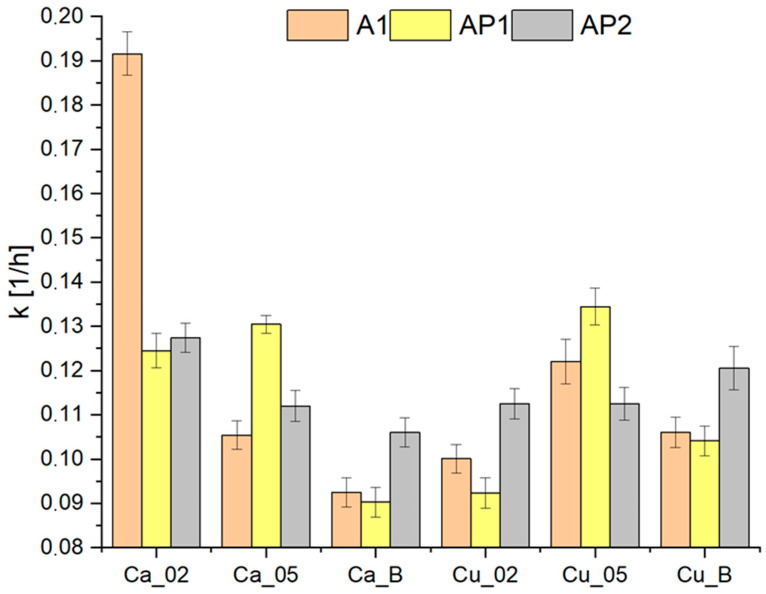
The drying rate constant of hydrogels—Newton model.

The values of the drying rate constant for the hydrogels using the Newton model are compiled in Figure 7. For crosslinking with Ca^2+^ ions, it can be stated that the highest value of the coefficient k was recorded for matrix A1 without the addition of PVA, around 0.19, which is the highest result for any of the remaining hydrogels. An increase in the concentration of Ca^2+^ ions leads to a decrease or stagnation of the drying rate constant of hydrogels, while the H_3_BO_3_ addition results in a significant reduction. As for the use of Cu^2+^ ions, the situation is different, as the lowest values of the constant k are present when using a concentration of 0.2 M. The higher concentration (0.5 M Cu^2+^) increases the constant k. The presence of H_3_BO_3_ also leads to growth, but smaller than by increasing the concentration of Cu^2+^ ions. Adding 2% PVA1 decreases the drying rate constant compared to matrix A1, although increasing the concentration to 10% results in its re-increase. The acquired drying rate constants correspond to the results from the comparison mass lost in Figure 4, indicating the correctness of the data and the translation of the calculated values of the drying rate constant.

**Figure 8:**
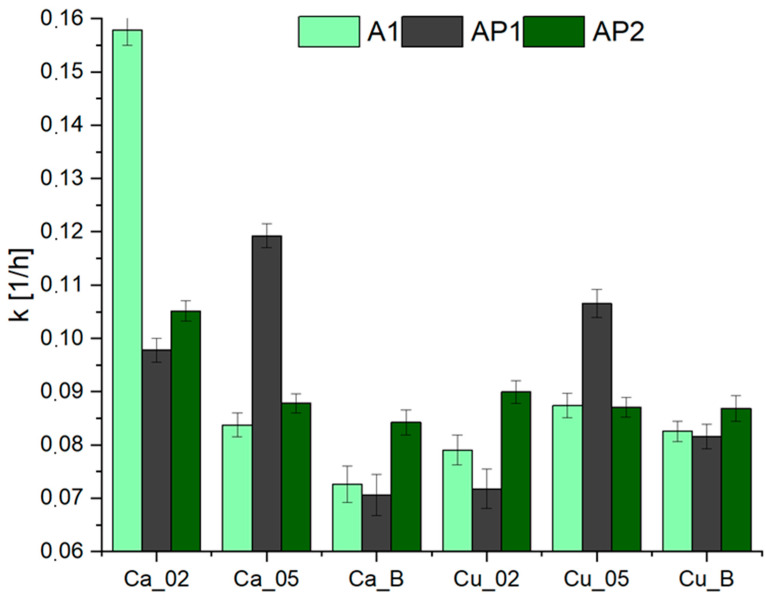
The drying rate constant of hydrogels—Page model.

**Figure 9:**
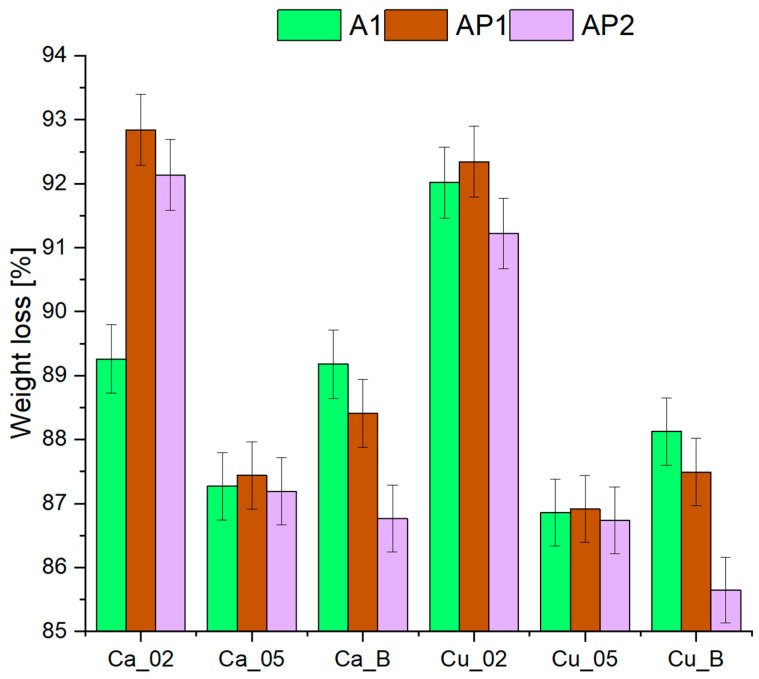
Weight loss of hydrogel samples—30 h drying.

Calculations were also performed for the constants of the Page model, as shown in Figure 8. The results are similar to those within the Newton model. Again, the highest value of the drying rate constant was acquired for hydrogel A1 crosslinked with 0.2 M Ca^2+^ ions. Once again, the lowest values of the drying rate constant were achieved for the matrix containing 2% PVA1, using low concentrations of Ca^2+^ and Cu^2+^. The addition of PVA also caused a significant decrease in the value of the drying rate constant when added to the crosslinking solution with H_3_BO_3_. It is worth noting that using 0.5 M crosslinking solutions increased the value of the drying rate constant in most of the tested samples. The results are not significantly different from those presented in Figure 6 and correspond to the laboratory results in Figure 6 and Figure 9. Differences between the model and experimental results may arise from measurement errors, as the values do not differ significantly, which means that the differences fall within the limits of statistical error.

The results from the drying kinetics studies indicate that the A1_Ca_02 group exhibited the weakest water retention properties (Figure 7) [33]. This fact may be due to the low concentration of Ca^2+^ ions, resulting in a less complete structure formed by achieving an insufficient bond number with alginate. The remaining hydrogels present much closer drying rate constants ranging from 0.095 to 0.120 1/h. Hydrogels containing 2% PVA1, when crosslinked with 0.2 M Cu^2+^, caused a decrease in the drying rate constant compared to the rest of the hydrogels, but increasing the concentration to 0.5 M accelerated it by about 30%. This may result from the displacement of PVA particles to the surface of the hydrogel, blocking water from evaporating in the outer layer of the acquired hydrogel for the 0.2 M solution [22,34]. The H_3_BO_3_ addition reduces the values of the drying constant, indicating that the inclusion of boron ions results in a denser hydrogel structure and PVA particles. In the case of SA hydrogels, PVA2, the constant values are higher, but the trends resulting from the crosslinking method are similar.

The conducted experimental studies (Figure 9) allow the estimation of the number of free spaces in the acquired structures. The observed decrease in mass loss in the samples of PVA1 crosslinked with Ca^2+^ and Cu^2+^ ions with the addition of H_3_BO_3_ indicates the achievement of a denser hydrogel structure [35]. This fact arises from the hydrogel’s property to form complex bonds between hydroxyl groups and boron ions. Higher concentrations of PVA resulted, in most cases, in an increase in mass loss, thereby leading to the formation of a more porous structure.

### 3.5. FTIR Analysis

**Figure 10:**
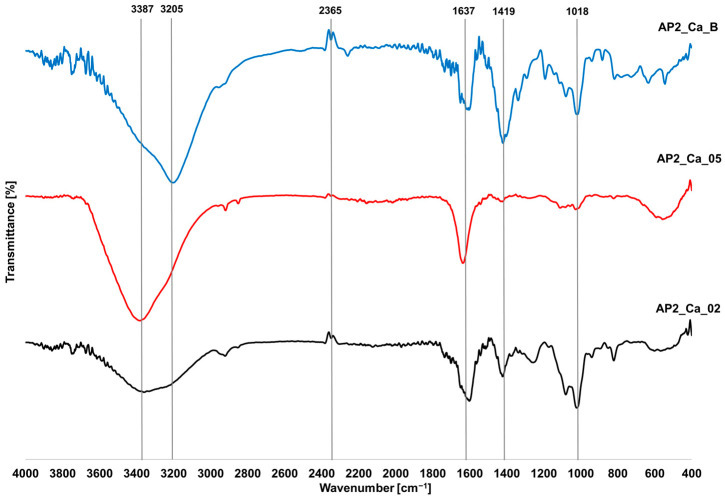
FTIR spectra of hydrogels based on SA/PVA2 cross-linked with Ca^2+^ ions.

**Figure 11:**
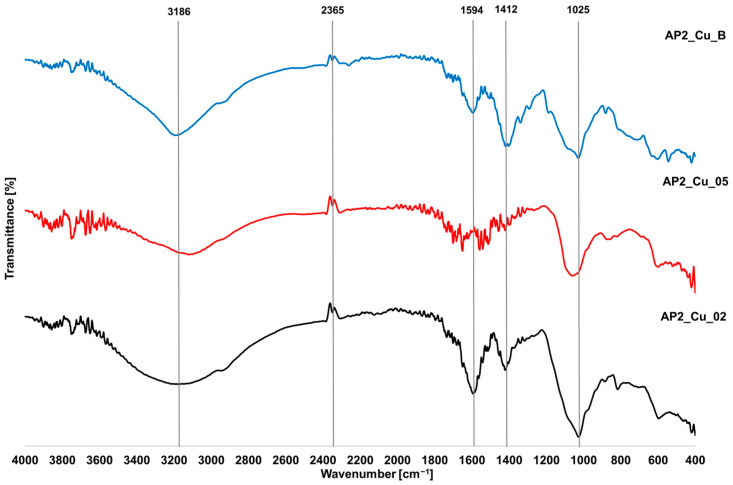
FTIR spectra of hydrogels based on SA/PVA2 cross-linked with Cu^2+^ ions.

FTIR analyses were conducted for each hydrogel containing PVA2 and are compiled in Figure 10 and Figure 11. The differences observed in spectra presented in Figure 10 resulted in the different concentrations of Ca^2+^ ions and the addition of boron ions. Three shared peaks are observed for all spectra at 1018, 1637, and 2365 cm^−1^. The difference occurs in the peak at the wavenumber of 3387 cm^−1^ for hydrogels crosslinked without the boron ions. The inclusion of H_3_BO_3_ caused a peak shift to 3205 cm^−1^. Hydrogels with boron ions exhibit a characteristic peak at the wavenumber of 1419 cm^−1^. When using 0.2 M Ca^2+^ ions, the described peaks are less pronounced. Figure 11 illustrates crosslinking using Cu^2+^ ions, which is analogous to the results acquired for crosslinking with Ca^2+^ ions. The peaks are in similar locations and reach similar wavenumber values. Peaks at wavenumbers 1412 and 1594 cm^−1^ are well visible for AP2_Cu_02 and AP2_Cu_B, while distortion occurs for the AP_Cu_05 spectrum. In the case of crosslinking with the addition of H_3_BO_3_, a characteristic peak occurs at the wavenumber of 3186 cm^−1^.

The FTIR spectra analysis facilitated the identification of functional groups within the tested materials, enabling comparisons of differences in hydrogel crosslinking methods. In the case of Ca^2+^ crosslinking in Figure 10, a shared peak at the wavenumber of 3387 cm^−1^ can be observed. It indicates the stretching of O-H bonds [36], where, for the AP2_Ca_02 hydrogel, a significant flattening of the peak compared to the AP2_Ca_05 sample can be noticed, indicating a smaller number of O-H groups. A smaller number of hydroxyl groups indicates a smaller amount of PVA chains. When H_3_BO_3_ was added, the mentioned peak shifted to 3205 cm^−1^ due to the presence of boron ions. The next peak occurred at the wavenumber of 2365 cm^−1^, which was shared for all tested hydrogels. This peak is associated with the presence of CO_2_ resulting from the methodology used in the research [37]. Next, joint peaks appear at the wavenumber of 1637 cm^−1^, indicating the presence of the C=O group in alginate [38]. Differences in the peak for individual crosslinking methods may arise from a lower degree of crosslinking with Ca^2+^ ions for the AP2_Ca_02 sample, where a lower concentration of the crosslinking agent resulted in a reduction in the degree of biopolymer interaction and, consequently, a smaller amount of carbonyl groups. As for the H_3_BO_3_ addition, a reaction between alginate and boron ions may occur. Confirmation of the influence of boron ions on the number of bonds associated with alginate is the apparent elongation of peaks at wavenumbers 1419 cm^−1^ and 1018 cm^−1^ associated with the presence of C-H and C-O-C bonds, respectively, occurring in the structure of alginate [38].

The influence of crosslinking with Cu^2+^ ions was also examined, as presented in Figure 11. Similar peaks were observed in the investigated structures but with different intensities. For the wavenumber of 3186 cm^−1^ associated with the O-H group, a significant weakening of the peak occurred outside the hydrogel with the addition of H_3_BO_3_, which may be related to the interaction of the crosslinking agent with the O-H group. This reduced the ability to absorb infrared waves or oxidize these groups. As for the C=O bonds (1594 cm^−1^) and C-H bonds (1412 cm^−1^), there is a weaker presence of the peak with increasing concentration of Cu^2+^ ions, which may be associated with the binding of Cu^2+^ ions to these groups, causing their flattening [39]. The same situation is noticeable for the C-O-C group (1025 cm^−1^) [40].

### 3.6. Thermogravimetric Analysis

**Figure 12:**
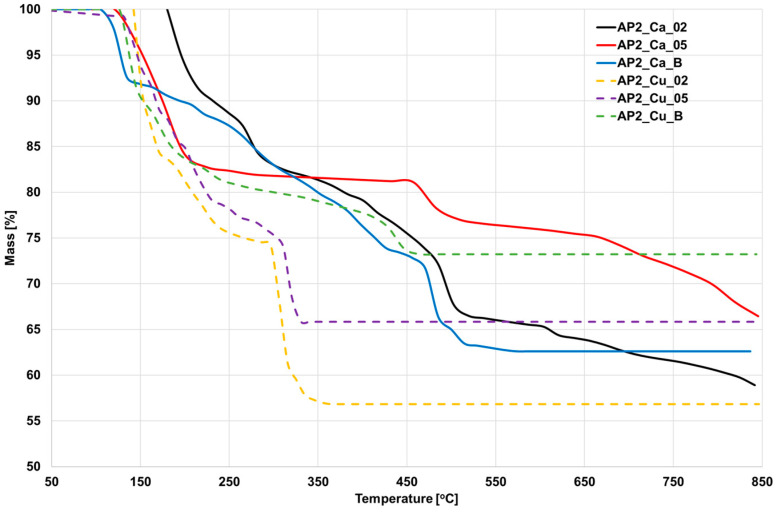
TGA diagram for AP2-based hydrogels.

The differences between the analyzed samples and thermogravimetric course are presented in Figure 12. The initial degradation of all hydrogels occurred in the range of 100–180 °C. The fastest loss was noted for AP2_Ca_05. Degradation continued up to a temperature of about 350 °C, where, subsequently, for samples crosslinked with Cu^2+^ ions without the boron ions, no stabilization occurred. As for the samples crosslinked with Ca^2+^ ions, a further decrease in mass occurred from a temperature of 475 °C, which was gradual. However, for sample AP_Cu_B, stabilization occurred. The mass loss for samples crosslinked with Cu^2+^ ions in the initial phase was similar to that of samples crosslinked with Ca^2+^ ions, but at a temperature of about 315 °C, a sharp decrease in mass emerged. The H_3_BO_3_ addition increased thermal stability.

Through the TGA analysis, approximately, an 5% mass loss was observed for all tested samples in the temperature range of 100–200 °C, most likely associated with moisture removal [29]. Subsequently, progressive degradation of alginate particles was observed, occurring within different temperature ranges depending on the crosslinking method [41]. Applying Cu^2+^ ions as a crosslinking medium resulted in 150–250 °C degradation, while crosslinking with Ca^2+^ ions caused degradation in the range of 175–300 °C. The highest mass loss due to alginate degradation was recorded for Cu^2+^ ions. Yet, it is worth noting that the H_3_BO_3_ addition affected the degradation of alginate particles differently. For Group AP2_Ca_B, it slowed down the degradation and extended it to the range of 200–400 °C, resulting in a more linear trend. In the case of Group AP2_Cu_B, the opposite effect was observed, with a rapid degradation compared to samples without H_3_BO_3_ presence. Subsequently, the degradation of PVA occurred in the temperature range of 400–550 °C in each of the tested hydrogels [42]. Depending on the concentration, there were variations in the thermal stability of the structures. Increasing the concentration of Ca^2+^ ions to 0.5 M resulted in a mass decrease to 75% of the initial value, while using a lower concentration of 0.2 M led to a reduction to 65%, indicating that a higher concentration of ions allows for acquiring a more thermally stable hydrogel. A similar trend was observed for Cu^2+^ ions, indicating a higher degree of crosslinking in the resulting structures. It is worth noting that crosslinking with Cu^2+^ ions resulted in a more significant mass loss than for hydrogels crosslinked with Ca^2+^ ions. This trend may be due to the formation of weaker thermal bonds. However, with increasing temperature, it can be observed that structures crosslinked with Ca^2+^ ions underwent further degradation, with a rapid decrease followed by stabilization at 66% for group AP2_Cu_05. In contrast, for hydrogels crosslinked with Ca^2+^ ions, the stabilized mass was below 60% of the initial mass. Additionally, the H_3_BO_3_ presence improved the thermal stability of both hydrogels crosslinked with Cu^2+^ and Ca^2+^ ions but also significantly slowed down the mass loss at each of the discussed stages. The formation of bonds between PVA molecules significantly impacted thermal stability.

## 4. Conclusions

The study detailed in this research sheds light on how the choice of materials for crosslinked hydrogels influences their physicochemical properties. Through the analysis of results from five different tests, several conclusions were drawn: Increasing the concentration of crosslinking ions in alginate molecules enhances both mechanical and thermal properties by fostering the formation of numerous bonds within the hydrogel structure. In comparison, copper ions create weaker and shorter bonds between alginate molecules than calcium ions, resulting in Alg-Cu hydrogels exhibiting lower thermal stability. Incorporating polyvinyl alcohol (PVA) with a molecular weight ranging from 13,000 to 23,000 g/mol improves the mechanical properties of most hydrogels, provided that their network is adequately bound. Moreover, introducing PVA with a molecular weight of between 30,000 and 70,000 g/mol and crosslinking using boric acid boosts mechanical strength and thermal stability by establishing multiple strong bonds between PVA molecules. The distinctive properties observed in hydrogels produced through different crosslinking methods and materials suggest promising avenues for future research. By modifying the structure of the hydrogel, its properties can be tailored to suit diverse applications across industries such as packaging, dressing, and food. This versatility expands the spectrum of potential applications and facilitates sustainable resource management by considering environmental factors.

## Figures and Tables

**Table 1:** Final concentrations of polymers in hydrogels.

Solution	SA (%)	PVA1 (%)	PVA2 (%)
A1	3.2	0	0
AP1	3.2	0.4	0
AP2	3.2	0	2

**Table 2:** Summary of analyzed hydrogel types.

	Crosslinking Solution					
Solution	CaCl_2_ (0.2 M)	CaCl_2_ (0.5 M)	CuSO_4_ (0.2 M)	CuSO_4_ (0.5 M)	CaCl_2_ (0.2 M) + H_3_BO_3_	CuSO_4_ (0.2 M) + H_3_BO_3_
A1	A1_Ca_02	A1_Ca_05	A1_Cu_02	A1_Cu_05	A1_Ca_B	A1_Cu_B
AP1	AP1_Ca_02	AP1_Ca_05	AP1_Cu_02	AP1_Cu_05	AP1_Ca_B	AP1_Cu_B
AP2	AP2_Ca_02	AP2_Ca_05	AP2_Cu_02	AP2_Cu_05	AP2_Ca_B	AP2_Cu_B

## Data Availability

Dataset available on request from the authors.
